# Fatal Dengue Hemorrhagic Fever in Adults: Emphasizing the Evolutionary Pre-fatal Clinical and Laboratory Manifestations

**DOI:** 10.1371/journal.pntd.0001532

**Published:** 2012-02-21

**Authors:** Ing-Kit Lee, Jien-Wei Liu, Kuender D. Yang

**Affiliations:** 1 Division of Infectious Diseases, Department of Internal Medicine, Kaohsiung Chang Gung Memorial Hospital, Kaohsiung, Taiwan; 2 Chang Gung University College of Medicine, Kaohsiung, Taiwan; 3 Department of Pediatrics, Show Chwan Memorial Hospital, Changhua, Taiwan; Pediatric Dengue Vaccine Initiative, United States of America

## Abstract

**Background:**

A better description of the clinical and laboratory manifestations of fatal patients with dengue hemorrhagic fever (DHF) is important in alerting clinicians of severe dengue and improving management.

**Methods and Findings:**

Of 309 adults with DHF, 10 fatal patients and 299 survivors (controls) were retrospectively analyzed. Regarding causes of fatality, massive gastrointestinal (GI) bleeding was found in 4 patients, dengue shock syndrome (DSS) alone in 2; DSS/subarachnoid hemorrhage, *Klebsiella pneumoniae* meningitis/bacteremia, ventilator associated pneumonia, and massive GI bleeding/*Enterococcus faecalis* bacteremia each in one. Fatal patients were found to have significantly higher frequencies of early altered consciousness (≤24 h after hospitalization), hypothermia, GI bleeding/massive GI bleeding, DSS, concurrent bacteremia with/without shock, pulmonary edema, renal/hepatic failure, and subarachnoid hemorrhage. Among those experienced early altered consciousness, massive GI bleeding alone/with uremia/with *E. faecalis* bacteremia, and *K. pneumoniae* meningitis/bacteremia were each found in one patient. Significantly higher proportion of bandemia from initial (arrival) laboratory data in fatal patients as compared to controls, and higher proportion of pre-fatal leukocytosis and lower pre-fatal platelet count as compared to initial laboratory data of fatal patients were found. Massive GI bleeding (33.3%) and bacteremia (25%) were the major causes of pre-fatal leukocytosis in the deceased patients; 33.3% of the patients with pre-fatal profound thrombocytopenia (<20000/µL), and 50% of the patients with pre-fatal prothrombin time (PT) prolongation experienced massive GI bleeding.

**Conclusions:**

Our report highlights causes of fatality other than DSS in patients with severe dengue, and suggested hypothermia, leukocytosis and bandemia may be warning signs of severe dengue. Clinicians should be alert to the potential development of massive GI bleeding, particularly in patients with early altered consciousness, profound thrombocytopenia, prolonged PT and/or leukocytosis. Antibiotic(s) should be empirically used for patients at risk for bacteremia until it is proven otherwise, especially in those with early altered consciousness and leukocytosis.

## Author Summary

Fatality rate and causes of fatality in dengue-affected patients greatly varied from one reported series to another. A better understanding of the clinical and laboratory manifestations of fatal patients with dengue hemorrhagic fever (DHF) is important in alerting clinicians of severe dengue and improving management. In a retrospective analysis of 10 adults who died of and 299 survived (controls) DHF, dengue shock syndrome (DSS) alone was found in only 20% of dengue-related death, while intractable massive gastrointestinal (GI) bleeding was found in 40%, and DSS with concurrent subarachnoid hemorrhage, intractable massive GI bleeding with concurrent bacteremia, bacterial sepsis/meningitis, and sepsis due to ventilator associated pneumonia each were found in 10%. Early altered consciousness (developed ≤24 h after hospitalization), GI bleeding/massive GI bleeding and concurrent bacteremia were significantly found among the deceased patients. Our data suggest that hypothermia, leukocytosis and bandemia at hospital presentation may be warning signs of severe dengue. Clinicians should be alert to the potential development of massive GI bleeding, particularly in patients with early altered consciousness, profound thrombocytopenia, prothrombin time prolongation and/or leukocytosis. Antibiotic(s) should be empirically used for patients at risk for bacteremia until it is proven otherwise, especially in those with early altered consciousness and leukocytosis.

## Introduction

Dengue is the most prevalent mosquito-borne viral infection in the world [1]. Clinically, dengue ranges from asymptomatic, nonspecific febrile illness, classic dengue, to dengue hemorrhagic fever/dengue shock syndrome (DHF/DSS) [1]. Fatality rate and causes of fatality in dengue-affected patients greatly varied from one report to another [1]–[13]. While DSS was the major cause of fatality in patients with dengue illness reported in some series [1]–[13], causes other than DSS were predominantly responsible for fatality reported in others [2], [10], [12]–[14]. However, only a small number of dengue-attributed mortality cases were included for analysis in each of these series [2], [8], [10]–[12]. A better description of the clinical and laboratory presentations of cases with fatal outcome may lead clinicians to an earlier recognition of the warning signs of severe dengue resulting in timely and improved management. To achieve this, the importance of continuous analysis of relevant findings in fatal patients from dengue-affected populations cannot be overemphasized.

Among the major dengue epidemics in Taiwan over the past 3 decades, a large dengue outbreak caused by DENV-1 occurred in 1987–1988 in southern Taiwan, followed by another one caused by DENV-2 in 2002 in the same geographic area [15]. During the 2002 dengue epidemic in southern Taiwan, more than 5000 dengue cases were reported, and most of them were DHF that developed in adults [15], [16]; of note, dengue-related fatality was found in 10 adults admitted at Kaohsiung Chang Gung Memorial Hospital (KSCGMH), a 2500-bed facility serving as a primary care and tertiary referral center in this area. In this study, we retrospectively compared the clinical and laboratory features of dengue-affected adults who turned out to be fatal and who survived, and analyzed the fatal dengue cases aiming at understanding the causes of mortality and clarifying the clinical and laboratory evolutions preceding mortality.

## Materials and Methods

### Ethics statement

The data in this work were analyzed anonymously, and the study was conducted with a waiver of patient consent approved by the Institutional Review Board of KSCGMH (Document No. 99-2671B).

### Patients and definitions

Patients with the diagnosis of dengue admitted to KSCGMH between June and December 2002 were potentially eligible for inclusion in this retrospective study. All clinically diagnosed dengue cases were serologically confirmed by at least one of the following criteria: (i) a positive reverse transcriptase-polymerase chain reaction (RT-PCR), (ii) a positive enzyme-linked immunosorbent assay for specific immunoglobulin M antibody for dengue virus in the acute-phase serum, and (iii) at least fourfold increase in dengue-specific hemagglutination inhibition titers in the convalescent serum when compared to that in acute-phase serum [17]. The diagnosis of DHF was established based on the presence of fever, hemorrhage, thrombocytopenia (<100×10^9^ cells/L) and clinical evidence of plasma leak (i.e., presence of hemoconcentration, pleural effusion, ascites and/or hypoalbuminemia) indicating increased vascular permeability [17]. Hemoconcentration referred to >20% increase in hematocrit calculated as: (maximum hematocrit - minimum hematocrit)×100/minimum hematocrit. The severity of DHF in serologically confirmed dengue patients was stratified based on the World Health Organization (WHO) criteria. Grade I referred to a positive tourniquet test result being the only hemorrhagic manifestation, while grade II referred to spontaneous bleeding. Grade III referred to a circulatory failure manifested by a rapid and weak pulse, as well as a narrowing pulse pressure (≦20 mmHg), whereas grade IV referred to a profound shock, with an undetectable pulse or blood pressure [17]. Grades III and IV of DHF were grouped as DSS [17].

All fatal DHF patients in this series resulted in dengue-related mortality which referred to the death that occurred within three weeks after hospitalization because of DHF. Hypothermia referred to a temperature <36°C detected at least twice from the ear drum of a dengue-affected patient. Massive gastrointestinal (GI) bleeding was defined as the passage of large amount of tarry or bloody stool coupled with hemodynamic instability and/or rapid decrease in hemoglobin level to ≦7.0 g/dL. Acute renal failure was defined as a rapid increase in serum creatinine (Cr) level ≧0.5 mg/dL when compared to that found at the patient's hospital presentation. Acute hepatic failure was defined as a raise in serum alanine aminotransferase (ALT) level ≧400 U/L (reference value, <40 U/L). Leukocytosis was defined as a peripheral white cell count >12000/µL. Bandemia referred to the presence of band-form granulocytes in the peripheral blood. Profound thrombocytopenia referred to a platelet count <20000/µL. Prolongation of prothrombin time (PT) was defined as a PT≥3 seconds than a control, and prolongation of activated partial thromboplastin time (APTT) as an APTT ≥20% than a control. Concurrent bacteremia was defined as a positive bacterial growth from blood that was sampled for culture within 72 h after the patient was hospitalized for dengue.

Demographic, clinical, laboratory and imaging information of the included DHF patients were retrieved from the retrospective review of their medical charts for analyses. Initial laboratory data referred to data detected from the dengue-affected patients upon their arrival at KSCGMH. Pre-fatal laboratory data were those detected from the blood specimens of the fatal patients sampled within the immediate 48 h before fatality.

### Statistical analysis

The 309 DHF patients included for analyses were separated into two groups: those who were fatal (fatal group, N = 10) and those survived (control group, N = 299). The survived patients were those with detailed information available. We compared the demographic, clinical, imaging characteristics and initial laboratory data of the fatal patients and those of the controls, as well as the pre-fatal laboratory data and the initial laboratory data of the fatal patients. Mann-Whitney *U* test was used in comparison of continuous variables, while the Fisher's exact test was used to in assessment of dichotomous variables. A 2-tailed *P*<0.05 was considered statistically significant.

## Results

### Description of demographics and clinical manifestations of fatal patients

A total of 714 adults with dengue illness were found at KSCGMH during the study period, and among them, 10 (8 men and 2 women; median age, 63.5 years [range, 33–78]) with DHF (7 grade II DHF and 3 DSS) turned out to be fatal, accounting for a dengue-related mortality rate of 1.3% (details are shown in Table S1). Of these fatal patients, the time lapses between dengue onset and hospital presentation ranged from 1 to 6 days (median, 2 days), between hospital presentation to fatality 2 to 18 days (median, 4.5 days), and between dengue onset to fatality 4 to 21 days (median, 7.5 days). With the exception of patient 2 in whom the dengue diagnostic test was carried out from the blood specimen collected on the day 3 of his hospitalization, all patients had their blood sampled for dengue diagnosis within 24 h after admission. The median from dengue onset to the definitive diagnosis made was 5 days (range, 4–11 days). Infection with DENV-2 in all fatal patients was confirmed by RT-PCR.

Manifestations indicating plasma leak in these fatal patients included hemoconcentration (patients 2, 5–10), presence of pleural effusion (patients 1, 3–6, 8 and 10) and hypoalbuminemia (patients 1, 2 and 4). Seven patients (patients 1–4, 6, 7 and 9) with grade II DHF experienced shock resulting from bacterial sepsis (patients 1 and 4), concurrent bacterial sepsis and massive GI bleeding (patient 9), and massive GI bleeding (patients 2, 3, 6, and 7). DSS alone was found in 3 (patients 5, 8 and 10) patients. Shock, regardless of cause, developed 1 to 16 days (median, 3 days) after their hospitalizations, and 4 and 17 days (median, 6.5 days) after dengue onset. Among the 3 DSS patients, DSS was recognized on day 3 (patients 8 and 10) and day 6 (patient 5) of their hospitalization, respectively. Patient 2 experienced 2 episodes of massive GI bleeding with hypovolemic shock on day 8 and day 16 of his hospital stay, respectively. Patient 4 with an underlying lung cancer suffered septic shock on day 15 of his hospitalization. The demographic, clinical and laboratory information of the fatal patients and controls is summarized in Tables 1 and 2.

**Table 1:** Demographic and clinical information of the fatal and control groups*.

Variable	Fatal groupN = 10	Control groupN = 299	*P*
**Demographic and clinical features**			
Median age, yrs (range)	63.5 (33–78)	55 (19–88)	0.219
Male	8 (80)	132 (44.1)	0.047
Underlying disease/condition†			
Diabetes mellitus	1 (10)	60 (20.1)	0.693
Hypertension	4 (40)	89 (29.8)	0.495
COPD	0	17 (5.7)	>0.99
Previous stroke	1 (10)	22 (7.4)	0.544
Chronic kidney disease	2 (20)	12 (4)	0.070
Parkinsonism	1 (10)	2 (0.6)	0.094
Solid tumor‡	1 (10)	3 (1)	0.124
Onset of dengue illness to arrival at hospital, median day (range)	2 (1–6)	3 (1–7)	0.055
Onset of dengue illness to shock, median day (range)	6.5 (4–17)	-	-
Onset of dengue illness to death, median day (range)	7.5 (4–21)	-	-
Admission to death, median day (range)	4.5 (2–18)	-	-
Hypovolemic shock due to massive GI bleeding	5§(50)	2 (0.7)	<0.001
DSS	4 (40)	7 (2.3)	<0.001
Concurrent bacteremia, n/N (%)	3/8 (37.5)	3/77 (3.9)	0.010
With shock, n/N (%)	2/8§ ¶ (25)	1**/77 (1.3)	0.022
Without shock††, n/N (%)	1/8 (37.5)	2/77 (3.9)	0.259
Pleural effusion, n/N (%)	7‡‡/10 (70)	81/212 (38.2)	0.054
Pulmonary edema, n/N (%)	3§§/10 (30)	6/212 (2.8)	0.005
Ascites, n/N (%)	0/3 (0)	64/160 (40)	0.280
Gallbladder swelling, n/N (%)	1/3 (33.3)	88/160 (55)	0.591
Acute renal failure, n/N (%)	8/8 (100)	6/298 (2)	<0.001
Acute hepatic failure, n/N (%)	4/7 (57.1)	5/114 (4.4)	<0.001
**Symptom/sign** ¶¶			
Fever (ear temperature >38°C)	9 (90)	283 (94.6)	0.437
Hypothermia (more than one ear temperature <36°C)	2*** (20)	0	0.001
Abdominal pain	2 (20)	154 (51.5)	0.059
Retro-orbital pain	3 (30)	36 (12)	0.119
Bone pain	6 (60)	174 (58.2)	>0.99
Myalgia	4 (40)	44 (14.7)	0.053
Cough	6 (60)	105 (35.1)	0.176
Headache	3 (30)	135 (45.2)	0.521
Rashes	1 (10)	92 (30.8)	0.292
Vomiting	4 (40)	48 (16.1)	0.069
Gum bleeding	1 (10)	75 (25.1)	0.460
Petechiae	4 (40)	190 (63.5)	0.183
GI bleeding	9 (90)	48 (16)	<0.001
Massive GI bleeding	5 (50)	2 (0.7)	<0.001
Subarachnoid hemorrhage	1 (10)	0	0.032
Early altered consciousness (<24 h after hospitalization)	4††† (40)	0	<0.001

*Data are number of patients (%), unless stated otherwise. DSS = Dengue shock syndrome; COPD = chronic obstructive pulmonary disease; GI = gastrointestinal; n/N = no. of patients/no. of patients with data available.

**†:** An individual patient might have more than one underlying disease/condition.

**‡:** One patient with lung cancer found in the fatal group; 2 patients with breast cancer and 1 with esophagus cancer found in the control group,.

**§:** One patient with hypovolemia had a concurrent *Enterococcus faecalis* bacteremia.

**¶:** Blood culture was available in 8 patients; one of them experienced *Klebsiella pneumoniae* meningitis and the other primary *E. faecalis* bacteremia, and shock was found in both.

**The patient had primary *Rosemonas* bacteremia.

**††:** Primary *K. pneumoniae* bacteremia was found in 1 patient the fatal group, and in 2 patients in the control group.

**‡‡:** Three patients concurrently had pulmonary edema.

**§§:** One lung cancer patient developed pulmonary edema in day 16 of his hospitalization.

**¶¶:** An individual patient might have more than one symptom and/or sign.

***One originally febrile patient developed hypothermia during hospital stay.

**†††:** Three (75%) patients experienced massive GI bleeding.

**Table 2:** Laboratory information of the fatal and control groups*.

Variable	Initial laboratory data(fatal group)† (A)	Initial laboratory data(control group)† (B)	Pre-fatal laboratory data(fatal group)‡ (C)	*P*(A vs. B)	*P*(C vs. A)
Leukopenia (WBC<3000/µL), n/N(%)	3/10 (30)	79/293 (27)	2/9 (22.2)	0.733	>0.99
Leukocytosis (WBC>12000/µL), n/N (%)	1/10 (10)	4/293 (1.4)	6/9 (66.7)	0.155	0.020
Bandemia§, n/N (%)	3/8 (37.5)	5/277 (1.8)	4/6 (66.7)	0.001	0.592
Median platelet count (µL) (range)	35000 (3000–157000)(N = 10)	93000 (1000–303000)(N = 299)	17000 (9000–108000)(N = 10)	0.088	<0.001
Platelet count <20000/µL), n/N (%)	2/10 (20)	62/299 (20.7)	6/10 (60)	>0.99	0.170
Median hemoglobin, g/dL (range)	11.7 (5.6–14.4) (N = 10)	13.1 (7.5–19.9) (N = 291)	8.9 (5.9–18.7) (N = 10)	0.091	0.261
Median hematocrit, % (range)	33.8 (16.6–41) (N = 10)	37.6 (16.7–57.2) (N = 282)	27.6 (16.6–54.8) (N = 10)	0.076	0.629
Prolongation of PT¶, n/N (%)	1/4 (25)	3/147 (2)	6/8 (75)	0.103	0.222
Prolongation of APTT**, n/N (%)	4/4 (100)	114/149 (76.5)	7/7 (100)	0.574	-
ALT >40 U/L (normal value <40 U/L), n/N (%)	5/5 (100)	74/114 (64.9)	3/4 (75)	0.167	0.444
AST>40 U/L (normal value <40 U/L), n/N (%)	6/7 (85.7)	125/161 (77.6)	5/5 (100)	>0.99	>0.99
Albumin <3.0 g/dL (normal value, 3.0–4.5 g/dL), n/N (%)	0/3 (0)	6/46 (13)	3/4 (75)	>0.99	0.143
Hyperkalemia (>6 meq/L), n/N (%)	0/10	0/158	1/6 (16.7)	>0.99	0.375

*WBC = white cell count; APTT = activated partial thromboplastin time; PT = prothrombin time; ALT = serum alanine aminotransferase; AST = serum aspartate aminotransferase; n/N = no. of patients/no. of patients with data available.

**†:** Data detected from specimen(s) sampled at patient's arrival at the hospital.

**‡:** Pre-fatal laboratory data were data detected from the blood specimens of fatal patients sampled within the immediate 48 h before fatality.

**§:** Bandemia referred to presence of band cell (immature white blood cell) in peripheral blood.

**¶:** Prolongation of PT was defined as a PT>3 seconds than that of control.

**Prolongation of APTT was defined an APTT>20% than that of control.

A variety of clinical manifestations were found in each of these 10 fatal patients (Table 1). The leading ones, in decreasing order, were fever (>38°C) (90%), GI bleeding (90%), pleural effusion (70%), bone pain and cough (each 60%). The previously reported early warning signs for severe dengue [11], [17], [18], namely, persistent vomiting was found in 4 (40%) patients, and sustained abdominal pain in 2 (20%). Pulmonary edema developed in 3 (patients 4, 5 and 8) patients; 2 of them with DSS experienced acute pulmonary edema emerged on day 5 (patient 5) and day 6 (patients 8) after dengue onset, respectively, while the other one (patient 4) with lung cancer and hypoalbuminemia (serum albumin, 1.4 g/dL [normal range, 3.0–4.5 g/dL]) experienced septic shock on day 15 of hospitalization (day 17 after dengue onset) thus receiving fluid resuscitation, and pulmonary edema was found on following day. Acute renal failure was found in all of 8 patients (patients 1, 2, 4–9) with data available, and acute hepatic failure in 4 (patients 1, 6–8) (57.1%) of 7 patients with data available. Concurrent bacteremia was noted in 3 (patients 1, 4 and 9) (37.5%) of 8 fatal patients from whom blood was sampled for bacterial culture within 72 h after their hospitalization (Table S1). Of these 3 bacteremic patients, one (patient 1) experienced *Klebsiella pneumoniae* meningitis, while the other two experienced primary *K. pneumonia* (patient 4) and *Enterococcus faecalis* bacteremia (patient 9), respectively. Of a total of 9 patients (patients 2–10) with GI bleeding, 4 (patients 3, 7, 9 and 10) (44.4%) developed GI bleeding at their arrival; 5 (patients 2, 3, 6, 7 and 9) (55.5%) experienced massive GI bleeding, and 3 (patients 3, 7 and 9) (33.3%) developed massive GI bleeding within 24 h after admission. Among the 5 patients with massive GI bleeding, *E. faecalis* bacteremia was found in one (patient 9); active bleeding was endoscopically found in another (patient 3) with a gastric ulcer, and in the other (patient 7) with hemorrhagic gastritis. Only patients 3 and 7 received endoscopic examination. Among the 5 patients with massive GI bleeding, acute renal failure developed in 2 (patients 2 and 9), and concurrent acute renal and hepatic failure in the other 2 (patients 6 and 7).

Of the 5 fatal patients with consciousness disturbance, 4 (patients 1, 2, 7 and 9) were found to developed altered consciousness within 24 hours and one (patient 5) in the day 4 of his hospital stay. All of these 5 patients had blood sampled for bacterial culture, and one of them (patient 1) had additional cerebrospinal fluid sampled for bacterial culture. Among the 4 patients with early altered consciousness, massive GI bleeding alone (patients 7), uremia and massive GI bleeding (patient 2), *E. faecalis* bacteremia and massive GI bleeding (patient 9), and *K. pneumoniae* meningitis and bacteremia (patient 1) each were found in one. Hypernatremia (serum sodium >170 meq/L [normal range, 134–148 meq/L]) was additionally found in patient 2 in day 8 of his hospital stay. Altered consciousness abruptly developed in patient 5 on day 4 of his hospitalization which resulted from subarachnoid hemorrhage disclosed by a brain computed tomography, and a cerebral angiographic study was deferred because of his critical condition and acute renal failure in particular; his blood culture for bacteria was negative, and although hyperkalemia (serum potassium, 7.9 meq/L [normal range, 3.6–5.0 meq/L]) was found on day 7, hemodialysis was not carried out as it was hemodynamically unstable. Neither hyperglycemia nor hypoglycemia was found in the 10 fatal patients in this series. Hyponatremia was not found in our series. Serum calcium level was not assayed in these fatal patients.

Hypothermia was noted in 2 (20%) patients (patients 6 and 9). One patient (patient 9) with hypothermia detected at arrival experienced concurrent primary *E. faecalis* bacteremia, while the other (patient 6) experienced abrupt change in temperature with a rapid switch from fever to hypothermia on day 4 of her hospital stay, and her blood bacterial culture was negative.

All patients experienced respiratory failure that necessitated mechanical ventilatory support. The mean time from patient's hospital presentation to starting mechanical ventilation was 3 days (range, 1–6 days), and the root causes of respiratory failure included massive GI bleeding (patients 3, 6, 7 and 9), sepsis (patient 1), DSS (patients 8 and 10), subarachnoid hemorrhage (patient 5), persistent drowsiness occurred within 24 h after hospitalization (patient 2) and lung cancer with pleural effusion (patient 4).

### Treatment for the 10 fatal patients

Intravenous fluid including 0.9% saline, Ringer's lactate, and 5% dextrose in 0.9% saline was administered at infusion rates ranging from 0.6 mL/Kg BW/h to 2.7 mL/Kg BW/h for the 10 fatal patients before development of shock and/or severe GI hemorrhage. In addition, transfusion of platelets and/or other blood component(s) (i.e., packed red blood cells and/or fresh frozen plasma) was given for these fatal patients. Intravenous fluid replacement and blood transfusion were detailed in Table S1.

Prior to shock development, intravenous fluid supplements with 0.9% saline for the 3 patients with DSS was 1.6 mL/Kg BW/h (patients 5), 1.3 mL/Kg BW/h (patient 8) and 0.8 mL/Kg BW/h (patient 10), respectively. Markedly elevated hemoglobin levels were found in patients with DSS on the day shock developed. Only platelet transfusion was given for these 3 patients before development of DSS. Among the 5 (patients 2, 3, 6, 7 and 9) patients with massive GI bleeding, intravenous fluid (0.9% saline or Ringer's lactate) supplement was infused at rates ranging from 1.4 mL/Kg BW/h to 2.5 mL/Kg BW/h before development of hypovolemic shock, and 2 to 8 units of packed red blood cells were transfused on the day the massive GI bleeding emerged (Table S1).

Because superimposing bacterial sepsis could not be excluded in these critically ill patients, all of them received intravenous antibiotic(s) within 72 h after admission. Upon hospitalization, the 3 patients (patients 1, 4 and 9) with concurrent bacteremia received empirical antibiotic(s) (i.e., ceftriaxone for patient 1, piperacillin and gentamicin for patient 4, and ceftriaxone and penicillin for patient 9) to which the subsequently isolated bacteria were susceptible in vitro.

### Causes of fatalities

When it comes to causes of fatality in these10 fatal patients, intractable massive GI bleeding with hypovolemic shock was found in 4 (40%) (patients 2, 3, 6 and 7), DSS alone in 2 (20%) (patients 8 and 10), while DSS with subarachnoid hemorrhage (patient 5), *K. pneumoniae* bacteremia and meningitis with septic shock (patient 1), sepsis due to mechanical ventilation associated pneumonia (patient 4), as well as concurrent *E. faecalis* bacteremia and intractable massive GI bleeding with shock (patient 9) each (10%) were found in one patient. None of the fatal patients underwent autopsy.

### Pre-fatal laboratory features in the fatal patients

Pre-fatal leukocytosis was found in 6 (patients 4, 5, 6, and 8–10) (66.7%) of the 9 patients (patients 1, 2, and 4–10) with data available, and bandemia in 4 (patients 1, 2, 5 and 7) (66.7%) of the 6 patients (patients 1, 2 and 4–7) in whom the differential count of peripheral white blood cells was available. Of the 6 patients with development of pre-fatal leukocytosis, 2 (patients 4 and 10) had leukopenia upon their arrival, 4 had their blood sampled for bacterial culture and *E. faecalis* bacteremia (patient 9) was found in one patient (25%), all experienced GI bleeding, and 2 (patients 6 and 9) (33.3%) developed massive GI bleeding. Prolongation of pre-fatal PT was found in 6 (75%) (patients 2, 4–6, 8 and 9) of the 8 (patients 1, 2, 4–6, and 8–10) patients with data available. Of note, all the 6 patients with pre-fatal PT prolongation developed GI bleeding, and of them, 3 (50%) experienced massive GI bleeding. Pre-fatal profound thrombocytopenia was found in 6 (60%) fatal patients (patients 1, 3, 5 and 8–10); of them, 5 (patients 3, 5 and 8–10) (83.3%) developed GI bleeding, and 2 (33.3%) (patients 3 and 9) experienced massive GI bleeding. Pre-fatal hyperkalemia was found in only 1 (patient 5) of the 6 (patients 2, 4, 5, 7–9) patients with data available.

### Comparisons of demographic, clinical and initial laboratory features between the fatal patients and controls, and comparisons of the pre-fatal and initial laboratory features of the fatal patients

Significant differences in demographics and clinical manifestations between fatal patients and controls included male gender (80% vs. 44.1%, *P* = 0.047), hypovolemic shock due to massive GI bleeding (50% vs. 0.7%, *P*<0.001), concurrent bacteremia (37.5% vs. 3.9%. *P* = 0.010), concurrent bacteremia with shock (25% vs. 1.3%, *P* = 0.022), DSS (40% vs. 2.3%, *P*<0.001), pulmonary edema (30% vs. 2.8%; *P* = 0.005), acute renal failure (100% vs. 2%; *P*<0.001), acute hepatic failure (57.1% vs. 4.4%; *P*<0.001), hypothermia (20% vs. 0%, *P* = 0.001), GI bleeding (90% vs. 16%, *P*<0.001), massive GI bleeding (50% vs. 0.7%; *P*<0.001), subarachnoid hemorrhage (10% vs. 0%, *P* = 0.032) and early altered consciousness (40% vs. 0%, *P*<0.001) (Table 1).

Significant higher proportion of bandemia (37.5% vs. 1.8%; *P* = 0.001) from initial laboratory data between the fatal patients and the controls, and significant higher proportion of pre-fatal leukocytosis (66.7% vs. 10%; *P* = 0.020) and lower pre-fatal platelet count (median, 17000 cells/µL vs. 35000 cells/µL; *P*<0.001) as compared to the initial laboratory data of the fatal patients were found (Table 2).

## Discussion

The time interval from the dengue onset to patients' arrival at KSCGMH between the fatal and control groups did not differ significantly (median, 2 days vs. 3 days; *P* = 0.055) (Table 1). The timing of admission in both the fatal and control groups allowed us to evaluate the critical evolutionary changes in the dengue-affected patients because critical events (e.g., dropped blood pressure and circulation collapse) usually occur between day 3 and day 7 of the disease course [17], [18].

Dengue case fatality rate was reported to vary from 0.5% to 5.0% [2]–[4], [7], [9], [10], [12]. However, once DSS developed, the case fatality may soar to as high as 12–44% [3]–[5]. Our series showed that of all dengue-related deaths, DSS alone accounted for only 20%, while intractable massive GI bleeding alone for 40%, and DSS with concurrent subarachnoid hemorrhage, intractable massive GI bleeding with concurrent bacteremia, bacterial sepsis with meningitis, and sepsis due to ventilator associated pneumonia each were responsible for 10%.

DSS is characterized by severe plasma leak that leads to rapidly developed shock, and timely volume replacement is the cornerstone of therapy for the affected patients [17]. Notably, the volumes of intravenous fluid supplement prior to the full blown development of DSS in the 3 patients (patients 5, 8, 10) in our series were obviously suboptimal [17], [18]; in spite of the subsequent fluid resuscitation and blood/blood component transfusion, they died of profound shock and multi-organ failure between day 6 and day 7 after the onset of illness. The pulmonary edema developed in the 2 patients (patients 5 and 8) with DSS on day 5 and day 6 after the dengue onset, respectively, was accompanied by a concurrent marked hemoconcentration (see Table S1 for details) suggested continuous fluid leakage from the intravascular compartment to the extravascular compartment and the lung alveolar space in particular, leading to profound shock and pulmonary edema. In contrast, the pulmonary edema developed on day 16 in patients 4 who had an underlying lung cancer with malignant pleural effusion obviously resulted from fluid overload.

The latest WHO scheme classified dengue in terms of clinical severity as severe dengue (i.e., presence of severe bleeding, severe plasma leak and/or severe organ involvement) or non-severe dengue; for practical reasons, patients with non-severe dengue were further separated into those with warning signs (i.e., abdominal pain, persistent vomiting, clinical fluid accumulation, mucosal bleeding, lethargy/restless, liver enlargement, and increase in hematocrit in concurrent with rapid decrease in platelet count) and those without them [18]. Severe dengue patients with aggravated plasma leak and/or bleeding necessitate aggressive fluid resuscitation and additional blood transfusion as necessary, while non-severe dengue patients with warning sign(s) require strict observation, appropriate medical intervention and intravenous hydration, as they are at high risk for evolving into critical phase–severe dengue [18]. In addition to the aforementioned ones, our data suggest that leukocytosis, bandemia and hypothermia may be warning signs of severe dengue. From the pathophysiological point of view, leukocytosis and/or bandemia indicates a superimposing bacterial infection and/or other stressful stimuli [19]. Our data suggested that massive GI bleeding (33.3%) and bacteremia (25%) be the major causes of DHF patients' pre-fatal leukocytosis. Significantly, leukocytosis was found in the deceased patients before their death, and bandemia was found at the fatal patients' hospital presentation (Table 2); the latter suggest that bandemia may be an early warning parameter of severe dengue.

Mucosal bleeding may occur in any patient with dengue, and if the patient remains stable with fluid resuscitation/replacement, the mucosal bleeding should be considered a minor one [18]. Minor mucosal bleeding in dengue patients often results from diapedesis of erythrocytes around blood vessels with little inflammatory reaction [20]. If major bleeding occurs, it is usually from the GI tract [2], [12], [21], [22], and one of the risk factors for major GI bleeding is the existence of a peptic ulcer [21], which is unfortunately not uncommonly develops in patients under stress [23]. One dengue series with 30 fatal dengue cases included disclosed that 80% of the fatal patients experienced GI bleeding, and severe bleeding with shock accounted for 30% of fatality [2]. The previously reported data [2], [12], [22] and ours suggest that even minor or moderate GI bleeding should be regarded as a warning sign of severe dengue and the patient in question needs close monitoring, as it potentially evolved into life-threatening intractable massive GI bleeding. Gastric ulcer and hemorrhagic gastritis each endoscopically found in one fatal patient with massive GI bleeding in our series raises the question of whether a H_2_-blocker or proton-pump inhibitor should be used in patients with severe DHF patients for prevention of massive GI bleeding. Further study is needed to answer this question.

Of note, massive GI bleeding was found in 75% of patients who experienced early altered consciousness (Table 1); 50% of patients with pre-fatal PT prolongation and 33.3% of patients with pre-fatal profound thrombocytopenia experienced massive GI bleeding. These data suggested that clinicians be alert to the potential development of severe GI bleeding when facing DHF patients with altered consciousness, and persistent PT prolongation and thrombocytopenia, and thereby initiate a timely management as necessary.

Abdominal pain and persistent vomiting, the previously reported clinical warning signs of severe dengue [11], [17], [18], did not differ between the fatal patients and controls in this series. In contrast, hypothermia significantly found in the fatal patients suggested that it should be considered a warning sign of severe dengue. Dengue-affected patients with hypothermia should therefore be intensively monitored, and aggressive workup is needed to clarify the potential cause(s) so that an effective treatment can be started timely.

It is noteworthy that 50% of our patients presented with early altered consciousness suffered concurrent bacterial sepsis (*K. pneumoniae* meningitis and *E. faecalis* bacteremia, respectively), highlighting the need for an immediate empirical antibiotic administration for dengue-affected patients with altered consciousness for the presumably superimposing bacterial sepsis until it is proven otherwise.

The bacteria (2 *K. pneumoniae* and 1 *E. faecalis* isolates) grew from culture of blood of 3 patients (two of them each with the underlying hypertension and lung cancer) sampled within 48 h after their admission were of normal intestinal flora. Our observation and previously reported concurrent bacteremia in patients with DHF caused by the members of Enterobacteriaceae [12], [24] suggested that DHF patients are vulnerable to bloodstream invasion by microbes from the intestinal tract where they normally inhabit. These findings are consistent with the development of portal of entry for bacteria in bowels by disintegration of intestinal mucosal barriers in DHF patients reported previously [25], [26]. Of the fatal bacteremic patients in this series, one patient with *K. pneumoniae* bacteremia and the other with simultaneous *K. pneumoniae* bacteremia and meningitis clearly experienced septic shock, while the shock in the patients who suffered massive GI bleeding and concurrently *E. faecalis* bacteremia might result from both hypovolemia and sepsis in view that *E. faecalis* has relatively low clinical virulence [27]. Nevertheless, our data suggest that when it comes to empirical use of antibiotic for suspicious concurrent bacteremia in dengue-affected patients, it is reasonable to cover bacteria from the intestinal tract.

It is not surprising that acute renal failure (100%) and acute hepatic failure (57.1%) exclusively developed in fatal DHF patients in our series, as severe plasma leakage, massive bleeding and/or profound shock would lead to tissue hypo-perfusion, potentially rendering acute renal failure and hepatic failure [16], [28], [29].

There are some limitations in the present study. First, the fatalities in this series may be biased by patients' severity resulting from patient selection and referral pattern in a single medical center. Second, the lack of a standardized treatment protocol for severe dengue cases might bias patients' clinical outcomes in this retrospective analysis; this study thus addressed the pre-fatal clinical and laboratory evolutions in the deceased DHF patients, but not the appropriateness of treatment for them. Third, the small number of fatal cases made the statistical power quite small.

In summary, our report highlights the causes of fatality other than DSS in patients with severe dengue, and suggested that in addition to those mentioned by the WHO 2009 scheme, hypothermia, leukocytosis, and bandemia may be warning signs of severe dengue. Early altered consciousness and GI bleeding/massive GI bleeding were significantly found among deceased DHF patients in this series. Dengue-affected patients should be closely monitored and appropriately treated once GI bleeding emerges, as it potentially evolved into massive GI bleeding; once massive GI bleeding develops, patients are at high risk for mortality, and this may be particularly true in patients with early altered consciousness, leukocytosis, profound thrombocytopenia and PT prolongation. Antibiotic(s) should be empirically added for patients at risk for developing concurrent bacteremia, especially in those with early altered consciousness and emergence of leukocytosis. Our data suggest that bandemia at hospital presentation may be a warning parameter for severe dengue, and monitoring the potential emergence of leukocytosis and persistence of thrombocytopenia may be helpful in evaluation of the progressive dengue severity. Further study is needed to confirm our observations. The findings of the suboptimal fluid resuscitations and blood/blood component transfusions in some of the fatal cases in this series underscores the importance of a timely effective volume replacement by fluid infusion and blood/blood component transfusion for patients with a severe dengue.

## Supporting Information

Table S1 Demographic, clinical and laboratory information of the 10 fatal patients with dengue hemorrhagic fever*.
(DOC)
