# Supplementary material for: Fatal Dengue Hemorrhagic Fever in Adults: Emphasizing the Evolutionary Pre-fatal Clinical and Laboratory Manifestations
Source: PLoS Negl Trop Dis. 2012 Feb 21;6(2):e1532. doi: 10.1371/journal.pntd.0001532 (PMC3283557; doi:10.1371/journal.pntd.0001532)
Supplement: Table S1 — Demographic, clinical and laboratory information of the 10 fatal patients with dengue hemorrhagic fever*. (DOC) [file pntd.0001532.s001.doc]

| **Table S1.** Demographic, clinical and laboratory information of 10 fatal patients with dengue hemorrhagic fever^*^ | | | | | | | | | | | |
| --- | --- | --- | --- | --- | --- | --- | --- | --- | --- | --- | --- |
|  | | | | | | | | | | | |
| **Patient no.** | **Age (yr)/ sex** | **Underlying disease/ condition** | **Severity of DHF** | **Blood culture result (timing of sampling blood, day)^† ‡^** | **Initial laboratory data (specimens sampled at arrival)** | **Pre-fatal laboratory data (timing of specimens sampled, day)^†‡^** | **Blood/blood component transfusion (day)^†^** **^‡§^** | **Intravenous fluid supplement (day)^†‡^** | **Complication(s) (day)^†‡^** | **Internal (day) between illness onset--arrival/ illness onset-- shock/admission--death** | **Cause of death** |
| 1 | 70/M | HTN | Grade II | *Klebisella pneumoniae* (D1) | WBC, 2800/μL  HB, 10.2 g/dL  PLT, 3000/μL  Cr, 2.0 mg/dL | WBC, 1900/μL (D2**^‡^**)  HB, 10.1 g/dL (D2**^‡^**)  PLT, 9000/μL (D2**^‡^**)  ALT, 1651U/L (D2**^‡^**)  AST, 1430U/L (D2**^‡^**)  Alb, 1.8 g/dL (D2**^‡^**)  APTT, 47.8 sec (control, 31.5) (D2**^‡^**)  Cr, 2.7 mg/dL (D2**^‡^**) | PLT 24 units (D1);  PLT 24 units + PRBC 2 units + FFP 4 units (D2**^‡^**) | 0.9% saline 1mL/kg BW/h (D1, D2**^‡^**); additional resuscitation with 0.9% saline 1000 mL (D2**^‡^**) | Drowsiness within 24h after hospitalization, endotracheal intubation, *K. pneumoniae* bacteremia and meningitis, septic shock, acute renal failure and acute hepatic failure (D2**^‡^**). | 2/4/2 | Septic shock |
| 2 | 68/M | HTN, DM, CKD and previous stroke | Grade II | Negative (D 1 and D 4) | WBC, 10300/μL  HB, 10.5 g/dL^¶^  PLT, 94000/μL  AST, 143 U/L  ALT, 69 U/L  Cr, 7.6 mg/dL^¶^ | WBC, 2300/μL (D18)  HB, 5.9 g/dL (D18)  PLT, 108000/μL (D18)  Alb, 1.5 g/dL (D17)  PT, 18.7 sec (control, 10.5) (D17)  Cr, 13.2 mg/dL (D18)  AST, 77 U/L (D17)  ALT, 33 U/L (D17) | PRBC 3 units (D8**^‡^**, D9 and D 10);  PLT 12 units (D11, D12 and D13);  PLT 12 units (D16**^‡^** and D17);  PRBC 3 units (D16**^‡^**) | 0.9% saline 0.6 mL/kg BW/h (D1-7); 0.9% saline 1.4 mL/kg BW/h (D8**^‡^**-D12); 0.9% saline 0.6mL/kg BW/h (D13-D15); 0.9% saline 2mL/kg BW/h (D16**^‡^**-D18); additional resuscitation with 0.9% saline 500mL (D16**^‡^**) | Persistent drowsiness within 24h after hospitalization, acute renal failure (D1), endotracheal intubation (D2), hypernatremia, massive GI bleeding with hypovolemic shock (D8**^‡^**) (improved after blood transfusion), and recurrent massive GI bleeding with hypovolemic shock (D16**^‡^**). | 2/10 and 18/18 | Intractable massive GI bleeding with hypovolemic shock |
| 3 | 49/M | None | Grade II | ND | WBC, 4300/μL**^‡^**  HB, 14.2 g/dL**^‡^**  PLT, 36000/μL**^‡^** | HB, 8.2 g/dL (D2)  PLT, 12000/μL (D2) | PLT 24 units (D1**^‡^**);  PRBC 2 units (D1**^‡^**) | 0.9% saline 2.5mL/kg BW/h (D1**^‡^**, D2); additional resuscitation with 0.9% saline 500mL (D1**^‡^**) | Massive GI bleeding (endoscopically found active gastric ulcer bleeding) with hypovolemic shock (D1**^‡^**), endotracheal intubation (D2). | 2/4/2 | Intractable massive GI bleeding with hypovolemic shock |
| 4 | 58/M | Lung cancer status post resection of right lower lobe | Grade II | *K. pneumoniae* (D1) | WBC, 2400/μL  HB, 13.2 g/dL  PLT, 29000/μL  Cr, 1.3 mg/dL  AST, 31 U/L | WBC, 20600/μL (D16)  HB, 10.1 g/dL (D16)  PLT, 105000/μL (D16)  APTT, 48 sec (control, 31) (D16)  PT, 15.7 sec (control, 10.7) (D16)  Alb, 1.4 g/dL (D16)  Cr, 2.0 mg/dL (D16) | PLT 12 units (D1);  FFP 4 units (D17) | 0.9% saline 1.5mL/kg BW/h (D1-D6); additional resuscitation with 0.9% saline 1000mL (D15**^‡^**) | Primary *K. pneumoniae* bacteremia (D1), right pleural effusion and endotracheal intubation (D6), GI bleeding and pneumonia (D14), shock (D15**^‡^**), acute renal failure and pulmonary edema (D16). | 2/17/17 | Septic shock due to ventilator associated pneumonia |
| 5 | 33/M | None | DSS | Negative (D1 ) | WBC, 3300/μL  HB, 13.0 g/dL  PLT, 79000/μL  APTT, 44.6 sec (control, 31.3)  Cr, 1.0 mg/dL  AST, 98 U/L  ALT, 110 U/L | WBC, 28300/μL (D6**^‡^**)  HB, 18.7 g/dL (D6**^‡^**)  PLT, 18000/μL (D6**^‡^**)  APTT, 60.2 sec (control, 30.4) (D6**^‡^**)  PT, >50 sec (control, 10.7) (D6**^‡^**)  AST, 6010 U/L (D6**^‡^**)  Cr, 10.3 (D7) | PLT 24 units (D4);  PLT 24 units + FFP 4 units (D6**^‡^**) | 0.9% saline 2.7mL/kg BW/h (D1); 0.9% saline 1.4 mL/kg BW/h (D2, D3); 0.9% saline 0.8mL/kg BW/h (D4, D5); 0.9% saline 1.6mL/kg BW/h (D6**^‡^**, D7); additional resuscitation with 0.9% saline 1000mL (D6**^‡^**) | Subarachnoid bleeding with drowsiness, acute renal failure, GI bleeding, pulmonary edema and endotracheal intubation (D4), hyperkalemia and shock (D6**^‡^**). | 1/7/7 | Subarachnoid bleeding and DSS |
| 6 | 46/F | ESRD post kidney transplant, and HTN | Grade II | ND | WBC, 6200/μL  HB, 8.3 g/dL^**^  PLT, 157000/μL  Cr, 1.8 mg/dL^**^  AST, 75 U/L | WBC, 20500/μL (D6**^‡^**)  HB, 6.4 g/dL(D6**^‡^**)  PLT, 62000/μL (D6**^‡^**)  APTT, 53.4 sec (control, 30.5) (D6**^‡^**)  PT, 18.6 sec (control, 10.5) (D6**^‡^**)  ALT, 1100 U/L (D6**^‡^**)  AST, 3423 U/L (D6**^‡^**)  Cr, 5.0 mg/dL(D6**^‡^**) | PLT 24 units (D2);  PLT 24 units (D3);  PLT 36 units + FFP 4 units (D4);  PRBC 2 units (D6**^‡^**) | 5 % dextrose in 0.9% saline 2mL/kg BW/h (D1- D3); 0.9% saline 1.6mL/kg BW/h (D4); 0.9% saline 2.5mL/kg BW/h (D5, D6**^‡^**); additional resuscitation with 0.9% saline 500mL (D6**^‡^**) | Hypothermia (D4), and massive GI bleeding with hypovolemic shock, acute renal failure, acute hepatic failure and endotracheal intubation (D6**^‡^**) | 1/7/6 | Intractable massive GI bleeding with hypovolemic shock |
| 7 | 78/M | None | Grade II | Negative (D1) | WBC, 3700/μL**^‡^**  HB, 14.4 g/dL**^‡^**  PLT, 15000/μL**^‡^**  APTT, 68 sec (control, 29.7) **^‡^**  PT, 14.2 sec (control, 10.3) **^‡^**  ALT, 670U/L**^‡^**  AST, 672U/L**^‡^**  Cr, 1.7 mg/dL**^‡^** | WBC, 7400/μL (D2)  HB, 8.5 g/dL (D2)  PLT, 53000/μL (D2)  Cr, 3.4 mg/dL (D2) | PLT 24 units + FFP 6 units + PRBC 8 units (D 1**^‡^**);  PLT 12 units + PRBC 2 units (D2) | 0.9% saline 1.5mL/kg BW/h (D1**^‡^**, D2), additional resuscitation with 0.9% saline 1500mL (D1**^‡^**) | Drowsiness within 24h and massive GI bleeding (endoscopically found active hemorrhagic gastritis bleeding) with hypovolemic shock, acute hepatic failure and endotracheal intubation (D1**^‡^**), and acute renal failure (D2) | 6/8/2 | Intractable massive GI bleeding with hypovolemic shock |
| 8 | 72/M | HTN | DSS | Negative (D3) | WBC, 10000/μL  HB, 14.3 g/dL  PLT, 25000/μL  APTT, 41.9 sec (control, 29.9)  Cr, 1.6 mg/dL  AST, 144 U/L  ALT, 67 U/L | WBC, 17700/μL (D4)  HB, 18.7 g/dL (D4)  PLT, 16000/μL (D4)  APTT, 59.0 sec (control, 29.5) (D4)  PT, 24.4 sec (control, 10.3) (D4)  Cr, 3.8 mg/dL (D4)  AST, 2673 U/L (D4)  ALT, 819 U/L (D4) | PLT 24 units (D1);  PLT 12 units (D2);  PLT 24 units + FFP 8 units (D3**^‡^**);  PLT 12 units + FFP 4 units (D4) | 0.9% saline 1.3mL/kg BW/h (D1, D2); additional resuscitation with 0.9% saline 2200mL (D3**^‡^**) and 1000mL (D4) | Shock, pulmonary edema and endotracheal intubation (D3**^‡^**), and acute renal failure, acute hepatic failure and GI bleeding (D4) | 3/6/5 | DSS |
| 9 | 70/M | Parkinsonism | Grade II | *Enterococcus faecalis* (D1**^‡^**) | WBC, 23800/μL**^‡^**  HB, 5 6 g/dL**^‡^**^††^  PLT, 34000/μL**^‡^**  APTT, 48 sec (control, 29.5) **^‡^**  Cr, 2.1 mg/dL**^‡^**^††^ | WBC, 20900/μL (D3)  HB, 8.5 g/dL (D3)  PLT, 15000/μL (D3)  APTT, 52.6 sec (control, 30.3) (D3)  PT, 20.9 sec (control, 10.6) (D3)  Cr, 2.9 mg/dL (D3) | PLT 12 units + PRBC 4 units (D1**^‡^**) | Ringer’s lactate 1.8mL/kg BW/h (D1**^‡^**); Ringer’s lactate 1mL/kg BW/h (D2); Ringer’s lactate 1.5mL/kg BW/h (D3-D4); additional resuscitation with 0.9% saline 500mL (D1**^‡^**), 600mL (D2) and 300mL (D3) | Drowsiness, hypothermia, *E. faecalis* bacteremia, massive GI bleeding, acute renal failure and shock (D1**^‡^**), endotracheal intubation (D3) | 3/5/4 | Sepsis and intractable massive GI bleeding with shock |
|  | 41/F | None | DSS | Negative (D2) | WBC, 1400/μL  HB, 8.4 g/dL  PLT, 54000/μL  AST, 151 U/L  ALT, 105 U/L  Cr, 0.7 mg/dL | WBC, 19500/μL (D3**^‡^**)  HB, 9.4g/dL (D3**^‡^**)  PLT, 15000/μL (D3**^‡^**)  APTT, 59.4 sec (control, 29.4) (D3**^‡^**) | PLT 24 units (D1);  PLT 12 units (D2);  PLT 12 units + PRBC 2 units (D3**^‡^**) | 0.9% saline 1.6 mL/kg BW/h (D1); 0.9% saline 0.8mL/kg BW/h (D2, D3**^‡^**); additional resuscitation with 0.9% saline 1000mL (D3**^‡^**) | GI bleeding (D1), endotracheal intubation and shock (D3**^‡^**). | 3/6/3 | DSS |

^*^ M = male; F = female; DHF = dengue hemorrhagic fever; D = day; DSS = dengue shock syndrome; PRBC = packed red blood cells; FFP = fresh frozen plasma; WBC = white blood cell; PLT = platelets; HB = hemoglobin; BW = body weight; Cr = creatinine; APTT = activated partial thromboplastin time; PT = prothrombin time; ALT = serum alanine aminotransferase; AST = aspartate aminotransferase; Alb = albumin; HTN = hypertension; GI = gastrointestinal; DM = diabetes mellitus; CKD = chronic kidney disease; ESRD = end stage renal disease; sec = second; h = hour; ND = no data

^†^Day 1 referred to the day the patient presented to the hospital.

^‡^The day in which shock developed.

^§^The volume of transfused platelets was 50 mL per unit, packed red blood cells 250mL per unit, and fresh frozen plasma 125mL per unit.

^¶^The baseline hemoglobin and serum creatinine levels found 8 months before this admission was 11.6g/dL and 2.1mg/dL, respectively.

^**^The baseline hemoglobin and serum creatinine levels found 3 months before this admission was 11g/dL and 1.6mg/dL, respectively.

^††^The baseline hemoglobin and serum creatinine levels found 2 months before this admission was 12.6 g/dL and 1.1mg/dL, respectively.
